# Fecal non-aureus Staphylococci are a potential cause of bovine intramammary infection

**DOI:** 10.1186/s13567-020-00761-5

**Published:** 2020-03-02

**Authors:** Ameline Wuytack, Anneleen De Visscher, Sofie Piepers, Freddy Haesebrouck, Sarne De Vliegher

**Affiliations:** 1 M-team and Mastitis and Milk Quality Research Unit, Department of Reproduction, Obstetrics, and Herd Health, Faculty of Veterinary Medicine, Ghent University, 9820 Merelbeke, Belgium; 2 Flanders Research Institute for Agriculture, Fisheries and Food (ILVO), Technology and Food Science, Agricultural Engineering, Burg. Van Gansberghelaan 115 Bus 1, 9820 Merelbeke, Belgium; 3 Department of Pathology, Bacteriology, and Avian Diseases, Faculty of Veterinary Medicine, Ghent University, 9820 Merelbeke, Belgium

## Abstract

The presence of non-aureus staphylococci (NAS) in bovine rectal feces has recently been described. Similar to other mastitis causing pathogens, shedding of NAS in the environment could result in intramammary infection. The objective of this study was to investigate whether NAS strains present in feces can cause intramammary infection, likely via teat apex colonization. During a cross-sectional study in 5 dairy herds, samples were collected from the habitats quarter milk, teat apices, and rectal feces from 25%, 10%, and 25% of the lactating cows, respectively, with a cow serving as the source of one type of sample only. Samples from clinical mastitis cases were continuously collected during the 1-year study period as well. The 6 most prevalent NAS species, *Staphylococcus* (*S.) chromogenes*, *S. cohnii*, *S. devriesei*, *S. equorum*, *S. haemolyticus*, and *S. hominis*, were further subtyped by random amplification of polymorphic deoxyribonucleic acid polymerase chain reaction (RAPD-PCR), when the same NAS species was present in the same herd in the three habitats. For *S. chromogenes*, *S. cohnii*, *S. devriesei*, and *S. haemolyticus*, the same RAPD type was found in rectal feces, teat apices, and quarter milk, indicating that fecal NAS can infect the mammary gland. For *S. hominis* and *S. equorum*, we were unable to confirm the presence of the same RAPD types in the three habitats.

## Introduction

Non-aureus staphylococci (NAS), a heterogeneous group of bacteria, are the most frequently identified bacteria in bovine milk samples worldwide [1–4]. They are also colonizing the teat apex of both lactating and dry cows [5, 6] as well as the teat canal [7] and other body parts [8]. We recently reported that *Staphylococcus* (*S.*) *agnetis*, *S. auricularis*, *S. chromogenes*, *S. cohnii*, *S. epidermidis*, *S. equorum*, *S. haemolyticus*, *S. hominis*, *S. kloosii*, *S. rostri*, and *S. xylosus* can also be isolated from rectal feces [9], indicating cows are shedding NAS into the environment which could eventually give rise to the establishment of intramammary infection (IMI), most likely after colonization of the teat apices. In that respect, *S. arlettae*, *S. auricularis*, *S. chromogenes*, *S. cohnii*, *S. devriesei*, *S. equorum*, *S. haemolyticus*, *S. hominis*, and *S. vitulinus* were recovered from rectal feces as well as from teat apices and quarter milk samples collected in the same herds (Wuytack et al. unpublished observations).

Intramammary infection typically occurs when bacteria enter the mammary gland through the teat canal. Quirk et al. [7] documented that teat canal colonization with *S. chromogenes*, *S. epidermidis*, *S. equorum*, *S. haemolyticus*, *S. hyicus*, and *S. simulans* was associated with IMI although strain-typing was not performed. NAS originating from the teat apices have been reported as a potential cause of IMI, as well. E.g. teat apex colonization with *S. chromogenes*, *S. equorum*, and *S. haemolyticus* has been described as a risk factor for NAS IMI based on species-level identification [10–12]. To date, a possible link between the presence of NAS species in feces and on teat apices and IMI has not been studied using a strain-typing approach.

Therefore, the purpose of this study was to investigate whether fecal NAS are potential causes of NAS IMI, likely via colonization of the teat apices.

## Materials and methods

### Herds, cows, and samples

Samples were obtained from 5 commercial dairy farms, all of them participating in the local dairy herd improvement program in Flanders with an interval of 4 to 6 weeks between 2 test-days (CRV, Arnhem, the Netherlands). The study was conducted during a 1-year period from March 2017 to March 2018. During the study period, herds had an average number of 75 lactating cows (ranging from 50 to 106) and a yearly milk production of 9304 kg milk (ranging from 8646 to 11 564). Bulk milk data were retrieved from MCC Flanders (Lier, Belgium). The geometric mean of the monthly bulk milk somatic cell count (SCC) was 106 000 cells/mL milk during the study (ranging from 77 000 to 148 000).

Quarter milk, teat apex, and rectal fecal samples were collected during a single cross-sectional sampling in each herd. Dairy cows were sampled once, either for quarter milk, teat apex swabs, or rectal feces. Quarter milk samples (total *n* = 334) were randomly obtained from approximately 25% of the lactating cows and collected using the guidelines of the National Mastitis Council (NMC) [13]. The SCC of the quarter milk samples was determined with a Direct Cell Counter (DCC, DeLaval, Tumba, Sweden) and used to stratify the quarters as healthy quarters (SCC ≤ 50 000 cells/mL milk) or as quarters with subclinical mastitis (SCC > 50 000 cells/mL). The teat apices (total *n* = 192) of a total number equal to approximately 10% of the lactating cows, were randomly sampled with a dry cotton swab (Copan, Novolab, Belgium) as described by De Vliegher et al. [14]. Both lactating and dry cows were sampled with a distribution of 1/3 and 2/3, respectively, and the teat apices of the lactating cows were sampled twice, before and after milking. Rectal fecal samples (total *n* = 80) were randomly obtained from approximately 25% of the lactating cows, based on the guarded sampling technique as used by Wuytack et al. [9]. A cow could serve as the source of one type of sample (quarter milk, teat apex, rectal feces) only.

During the entire study period, quarter milk samples of all clinical mastitis cases (total *n* = 103) were collected secundum artem by the producers.

All samples were transported under cooled conditions (4 °C) to the Mastitis and Milk Quality Research Laboratory (Faculty of Veterinary Medicine, Ghent University, Merelbeke, Belgium).

### Bacterial isolates

Quarter milk samples were plated with a 0.01 mL loop. Rectal feces were first diluted [1 g in 9 mL of 0.9 physiological salt solution (9 g NaCl/L, Eurovet, Bladel, The Netherlands)] and then plated with a 0.01 mL loop. All milk samples were streaked on aesculin blood agar (Oxoid, Aalst, Belgium), MacConkey agar (Oxoid), and the semi-selective agar, mannitol salt agar (MSA, Chapman medium, Oxoid) for the recovery of NAS isolates. Samples from teat apices or rectal feces were only streaked on MSA to prevent overgrowth of bacteria which are frequently observed [15, 16]. All plates were examined at 24 and 48 h after incubation at 37 °C. All phenotypically different colonies (≥ 1 colony) were analyzed following the NMC procedure guidelines for bacteriological identification [13]. Matrix-assisted laser desorption/ionization time-of-flight mass spectrometry (MALDI-Tof) was used to identify the *Staphylococcus* isolates at the species level [17]. When no identification with the MALDI-Tof could be obtained, *rpoB*, and/or 16S rRNA gene sequencing was performed [18]. All isolates originating from the MSA plates and phenotypically identified as *Staphylococcus* spp. were stored at −80 °C.

### Random amplification of polymorphic DNA-analysis

A selection of isolates of the 6 most prevalent NAS species (*S. chromogenes*, *S. cohnii*, *S. devriesei*, *S. equorum*, *S. haemolyticus*, and *S. hominis*) was analyzed for further subtyping by random amplification of polymorphic DNA polymerase chain reaction (RAPD-PCR). The selection was based on the following criteria: the isolates had to (1) originate from the same herd, (2) belong to the same NAS species, and (3) be present in the 3 different habitats (quarter milk, teat apices, and rectal feces). If more than 20 teat apex isolates from the same NAS species were available, only 10 isolates were selected. In that case, teat apex isolates originating from different quarters and cows and yielding the highest number of colonies were selected.

Deoxyribonucleic acid (DNA) was extracted with the use of the commercial DNeasy Blood and Tissue Kit (Qiagen, Hilden, Germany) following the manufacturer’s instructions. All sets of extractions were run with a negative control consisting of kit reagents with no sample to control for kit or environmental contamination. Aliquots of the DNA samples were stored at −20 °C until further analysis.

The RAPD-PCR was performed as described by Wuytack et al. [9], using the primer D11344 with the following PCR conditions: 4 cycles of 94 °C at 5 min, 36 °C at 5 min, and 72 °C at 5 min and 30 cycles of 94 °C at 1 min, 36 °C at 1 min, and 72 °C at 2 min. Isolates belonging to the same NAS species, originating from the same farm were analyzed together, in the same PCR run. Images were imported in BioNumerics version 7.6.3 (Applied Maths, Sint-Martens-Latem, Belgium) and analyzed with the Dice similarity coefficient and the unweighted pair group method with arithmetic mean (UPGMA) with an optimization set at 0.5% and position tolerance at 1.0%. Isolates with the same banding pattern, number, and size of bands, were considered the same RAPD type and given an arbitrary lower case letter.

## Results

The isolates used in the current study are part of a larger study (Wuytack et al., unpublished) comparing the NAS distribution in milk, on teat apices, and in rectal feces in multiple commercial dairy herds, comprising a total of 1228 isolates (365 from milk, 830 from teat apices, and 33 from rectal feces, respectively). Nine NAS species were present in quarter milk, on teat apices, and in rectal feces in the same herd, namely *S. arlettae*, *S. auricularis*, *S. chromogenes*, *S. cohnii*, *S. devriesei*, *S. equorum*, *S. haemolyticus*, *S. hominis*, and *S. vitulinus.* Other species were isolated from only one habitat, namely *S. hyicus* and *S. warneri* in quarter milk and *S. agnetis*, *S. kloosii*, *S. pettenkoferi*, and *S. rostri* on teat apices. None of the NAS species were solely present in rectal feces. In total, 194 NAS isolates (70 from quarter milk, 103 from teat apices, and 21 from rectal feces) from the larger collection of 1228 isolates belonging to the species *S. chromogenes*, *S. cohnii*, *S. devriesei*, *S. equorum*, *S. haemolyticus*, and *S. hominis* were selected for RAPD-PCR (Table 1).

**Table 1:** **Overview of the NAS isolates from milk (M), teat apices (T), and rectal feces selected for RAPD-PCR, divided over the 5 herds and 3 habitats.**

NAS species	Herd 1			Herd 2			Herd 3			Herd 4			Herd 5			Total
	M	T^1^	F	M	T^1^	F	M	T^1^	F	M	T^1^	F	M	T^1^	F	
*S. chromogenes*	13	8	1													22
*S. cohnii*				4	11	2										17
*S. devriesei*										9	11	2				22
*S. equorum*				3	8	1										12
*S. haemolyticus*	10	11	2										4	11	1	39
*S. hominis*	6	11	1	2	19	8	5	7	1	14	6	2				82

^1^If more than 20 isolates originating from the teat apices were available, a selection of only 10 isolates was made by selecting isolates originating from different quarters and cows and yielding the highest number of colonies.

**Figure 1:**
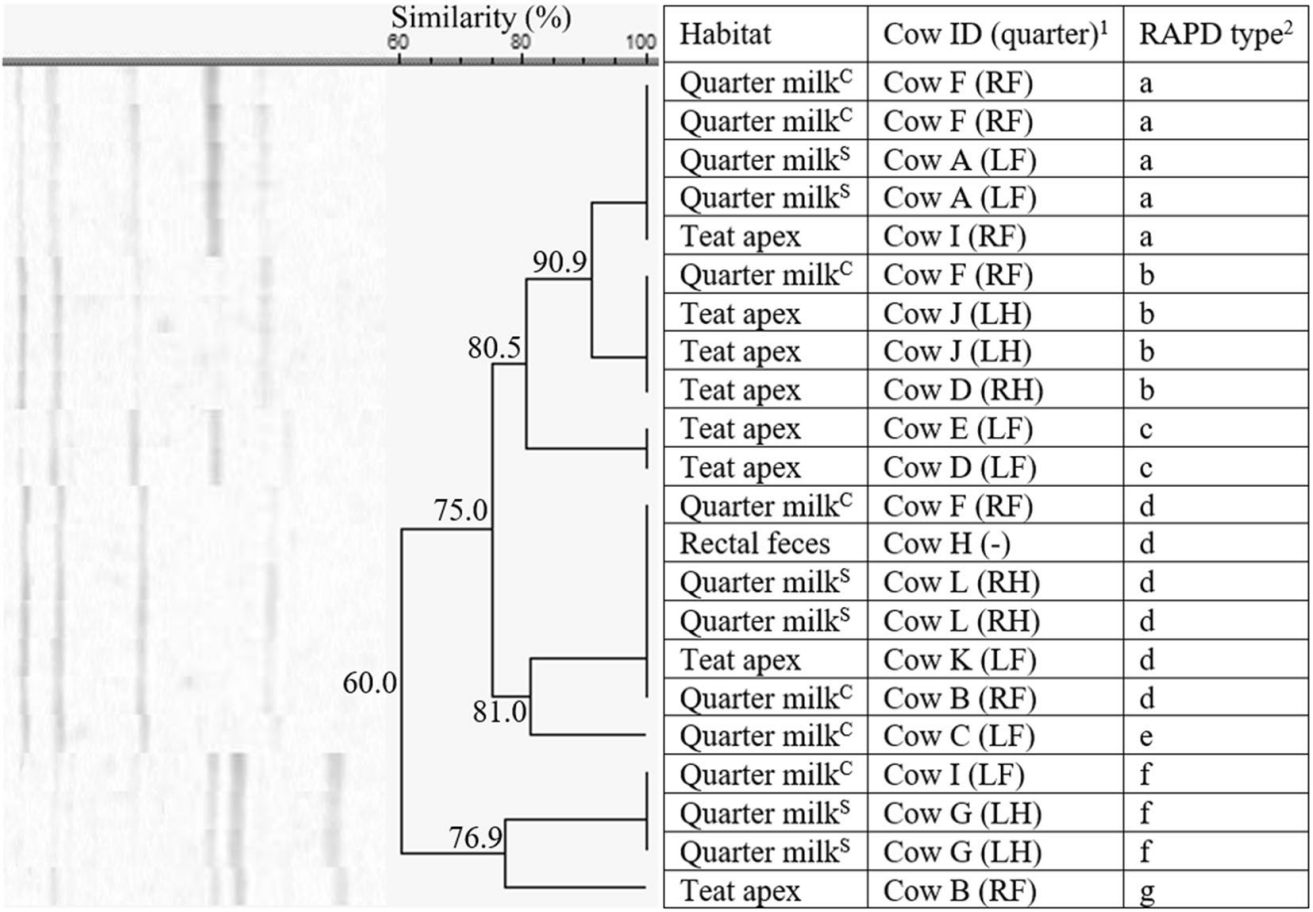
**Dendrogram of the RAPD fingerprints of 22*Staphylococcus chromogenes*isolates, originating from herd 1.**^1^Cows were assigned an arbitrary upper case letter, independently of the other dendrograms. ^2^RAPD types were assigned an arbitrary lower case letter based on the clustering. ^H^Quarter milk samples with a SCC ≤ 50 000 cells/mL milk (healthy). ^S^Quarter milk samples with a SCC > 50 000 cells/mL milk (subclinical mastitis). ^C^Quarter milk samples from a quarter with clinical signs of mastitis (clinical mastitis).

Among the 22 *S. chromogenes* isolates, originating from herd 1 only, 7 different fingerprints (a-g, 60% similarity) were identified (Figure 1). Five RAPD types (a, b, c, d, and f) were identified more than once. RAPD type d (*n* = 6) was isolated from all the habitats, i.e. quarter milk (as cause of both subclinical and clinical mastitis, *n* = 4), teat apices (*n* = 1), and rectal feces (*n* = 1). On the other hand, type f was only detected in milk, as cause of both subclinical and clinical mastitis. In cow F, multiple isolates of *S. chromogenes* (*n* = 4) were found in the same quarter (clinical mastitis) over a time period of 32 days, belonging to 3 different RAPD types.

**Figure 2:**
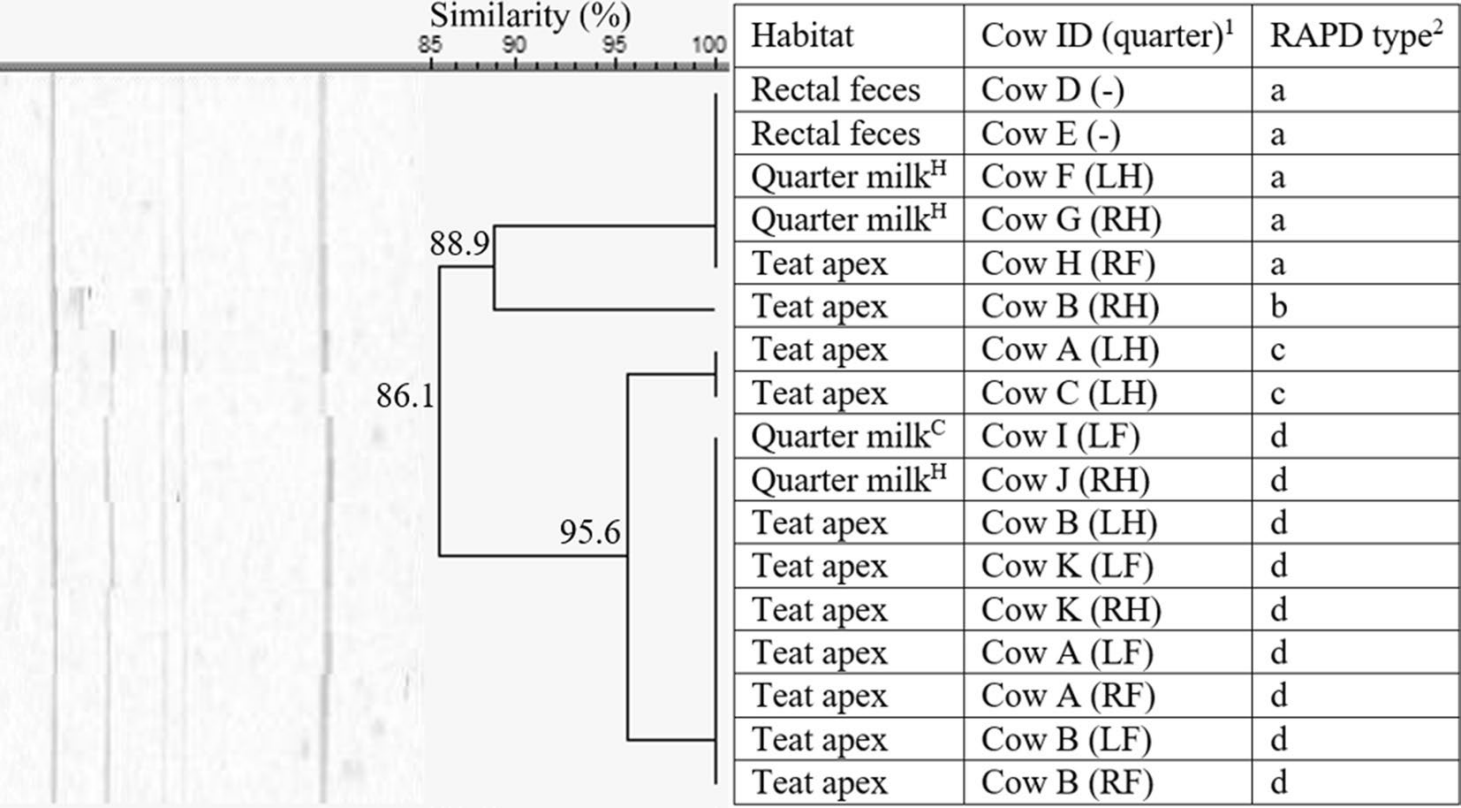
**Dendrogram of the RAPD fingerprints of 17*Staphylococcus cohnii*isolates, originating from herd 2.**^1^Cows were assigned an arbitrary upper case letter, independently of the other dendrograms. ^2^RAPD types were assigned an arbitrary lower case letter based on the clustering. ^H^Quarter milk samples with a SCC ≤ 50 000 cells/mL milk (healthy). ^S^Quarter milk samples with a SCC > 50 000 cells/mL milk (subclinical mastitis). ^C^ Quarter milk samples from a quarter with clinical signs of mastitis (clinical mastitis). ^C^Quarter milk samples from a quarter with clinical signs of mastitis (clinical mastitis).

Seventeen *S. cohnii* isolates originating from herd 2 only, were divided over 4 RAPD types (a–d, 86.1% similarity, Figure 2), with a, c, and d being identified more than once. Isolates of RAPD type a (*n* = 5) were isolated from quarter milk with a low SCC, teat apices, and rectal feces from 5 different cows, whereas type b and c were only detected on teat apices. RAPD type d (*n* = 9) was mainly found on either 2 or 3 teat apices from 3 different cows and 2 quarter milk samples, but not in feces (Figure 2).

**Figure 3:**
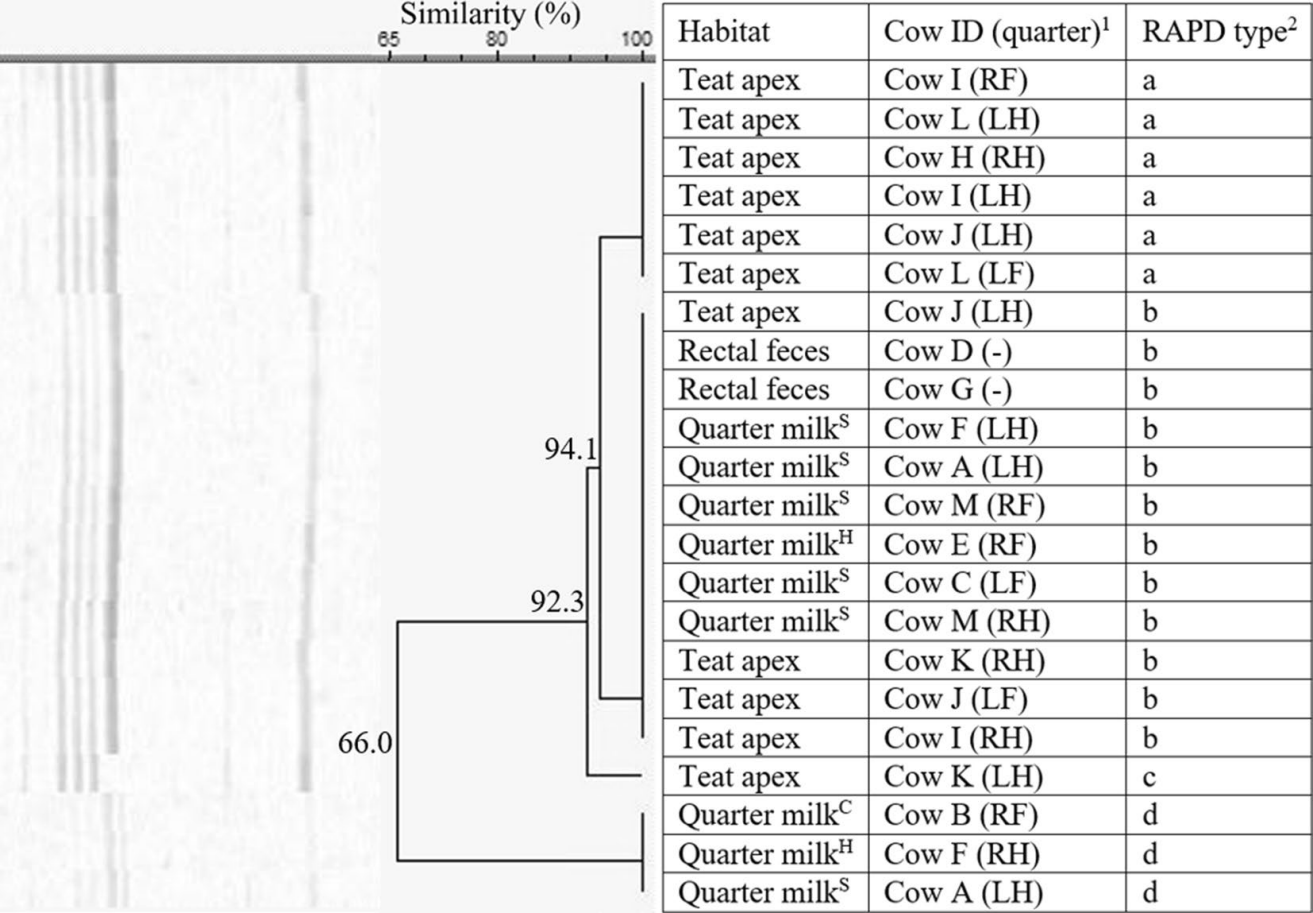
**Dendrogram of the RAPD fingerprints of 22*Staphylococcus devriesei*isolates, originating from herd 4.**^1^Cows were assigned an arbitrary upper case letter, independently of the other dendrograms. ^2^RAPD types were assigned an arbitrary lower case letter based on the clustering. ^H^Quarter milk samples with a SCC ≤ 50 000 cells/mL milk (healthy). ^S^Quarter milk samples with a SCC > 50 000 cells/mL milk (subclinical mastitis). ^C^ Quarter milk samples from a quarter with clinical signs of mastitis (clinical mastitis).

Random amplification of polymorphic DNA divided the 22 isolates of *S. devriesei* from herd 4 in 4 RAPD types (a–d, 66% similarity, Figure 3), with a, b, and d being identified more than once. RAPD type a was only colonizing teat apices whereas b was isolated 12 times from the three habitats, i.e. quarter milk both with a low and an elevated SCC, teat apices, and rectal feces. Cow A and J harbored multiple RAPD types in one quarter milk and one teat apex sample, respectively (Table 2).

**Table 2:** **Overview of the random amplification of polymorphic DNA (RAPD)-typing results for all samples harboring phenotypically different isolates from the same NAS species.**

NAS species	Herd	Cow^1^	Quarter	N_isolates_	Habitat	RAPD type^2^
*S. chromogenes*	Herd 1	Cow B	RF	2	QM^C^, TA	d, g
		Cow F	RF	4	QM^C^	a, b, d^3^
*S. devriesei*	Herd 4	Cow A	LH	2	QM^S^	b, d
		Cow J	LH	2	TA	a, b
*S. equorum*	Herd 2	Cow I	LF	2	TA	d, e
*S. haemolyticus*	Herd 1	Cow H	RF	2	QM^H^	c, d
		Cow K	LH	2	TA	g
*S. hominis*	Herd 1	Cow B	LH	2	QM^C^	c, d
		Cow G	LF	2	TA	e, e
		Cow H	RF	4	TA	e, f
		Cow H	RH	3	TA	f
	Herd 2	Cow C	LF	3	TA	b, e
		Cow E	–	2	RF	a, e
		Cow F	–	3	RF	c, d
		Cow G	–	2	RF	e, e
		Cow I	RH	2	TA	g, m
		Cow J	RH	3	TA	h, l
	Herd 3	Cow G	LF	3	TA	a, g
		Cow G	LH	2	TA	a
	Herd 4	Cow C	LH	3	QM^S^	c
		Cow K	LH	3	TA	b, c, i
		Cow K	LF	2	TA	e, g

^1^Cows were assigned an arbitrary upper case letter, independently of the other dendrograms.

^2^RAPD types were assigned an arbitrary lower case letter based on the clustering.

^3^Cow F (RF quarter) had 2 clinical mastitis cases with an interval of 32 days. *Staphylococcus chromogenes* RAPD type a was isolated from the first clinical mastitis case and *S. chromogenes* RAPD type b and d were isolated from the second clinical mastitis case.

^H^Quarter milk samples with a SCC ≤ 50 000 cells/mL milk (healthy).

^S^Quarter milk samples with a SCC > 50 000 cells/mL milk (subclinical mastitis).

^C^Quarter milk samples from a quarter with clinical signs of mastitis (clinical mastitis).

^C^Quarter milk samples from a quarter with clinical signs of mastitis (clinical mastitis).

**Figure 4:**
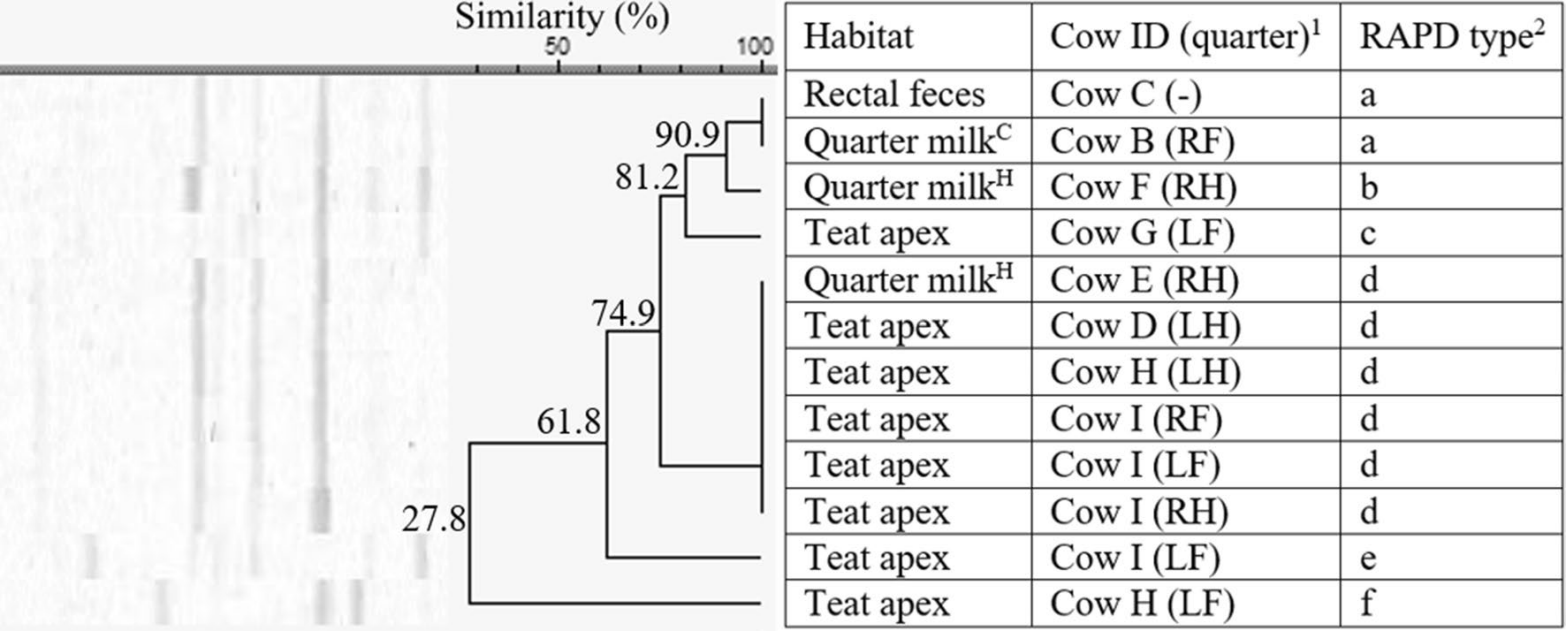
**Dendrogram of the RAPD fingerprints of 12*Staphylococcus equorum*isolates, originating from herd 2.**^1^Cows were assigned an arbitrary upper case letter, independently of the other dendrograms. ^2^RAPD types were assigned an arbitrary lower case letter based on the clustering. ^H^Quarter milk samples with a SCC ≤ 50 000 cells/mL milk (healthy). ^S^Quarter milk samples with a SCC > 50 000 cells/mL milk (subclinical mastitis). ^C^Quarter milk samples from a quarter with clinical signs of mastitis (clinical mastitis).

The 12 *S. equorum* isolates originating from herd 2 only, were divided in 6 RAPD types (e–f, 27.8% similarity, Figure 4), with a and d being identified more than once. None of the RAPD types was isolated from all the habitats. Type a (*n* = 2) was found in quarter milk (clinical mastitis) and rectal feces while type d (*n* = 6) was found in quarter milk from healthy quarters and on teat apices. All 4 teat apices of cow I were positive for *S. equorum,* belonging to RAPD type d or e.

**Figure 5:**
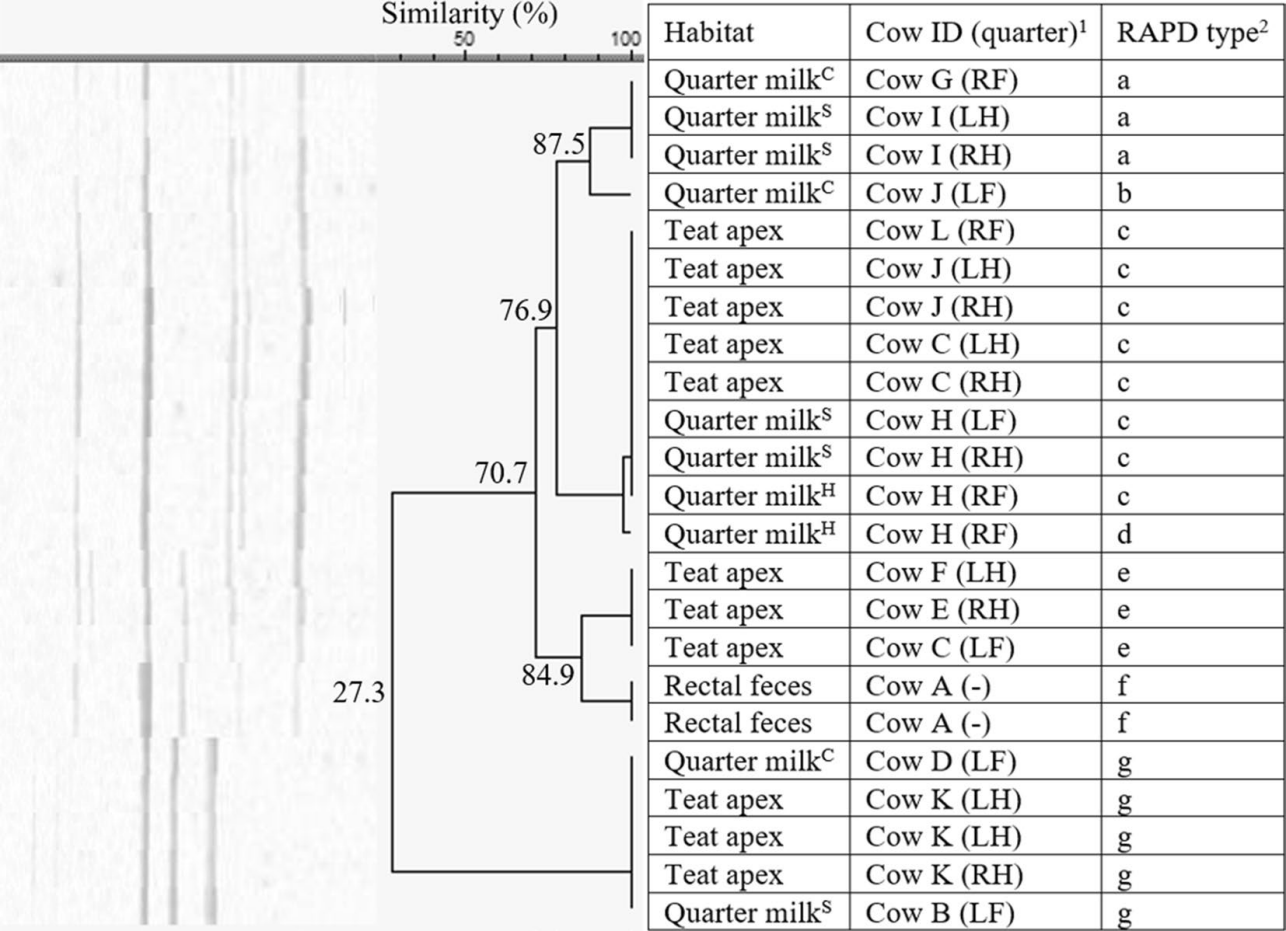
**Dendrogram of the RAP fingerprints of 23*Staphylococcus haemolyticus*isolates, originating from herd 1.**^1^Cows were assigned an arbitrary upper case letter, independently of the other dendrograms. ^2^RAPD types were assigned an arbitrary lower case letter based on the clustering. ^H^Quarter milk samples with a SCC ≤ 50 000 cells/mL milk (healthy). ^S^Quarter milk samples with a SCC > 50 000 cells/mL milk (subclinical mastitis). ^C^Quarter milk samples from a quarter with clinical signs of mastitis (clinical mastitis).

**Figure 6:**
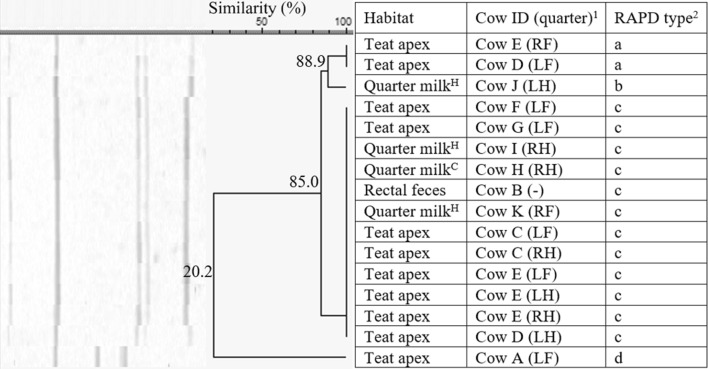
**Dendrogram of the RAPD fingerprints of 16*Staphylococcus haemolyticus*isolates, originating from herd 5.**^1^Cows were assigned an arbitrary upper case letter, independently of the other dendrograms. ^2^RAPD types were assigned an arbitrary lower case letter based on the clustering. ^H^Quarter milk samples with a SCC ≤ 50 000 cells/mL milk (healthy). ^S^Quarter milk samples with a SCC > 50 000 cells/mL milk (subclinical mastitis).

*Staphylococcus haemolyticus* was isolated from all habitats from two herds, i.e. herd 1 (*n* = 23, 27.3% similarity), with a, c, e, f, and g being identified more than once, and herd 5 (*n* = 16, 20.2% similarity), with a and c, being identified more than once. In herd 1, the isolates belonged to 7 different RAPD fingerprints (a–g, Figure 5). The isolates originating from the rectal feces (*n* = 2) belonged to the same RAPD type f, originating from cow A. RAPD type f was not retrieved from quarter milk or teat apices. The *S. haemolyticus* isolates originating from quarter milk or teat apices were divided in 6 other RAPD types. Cow H and I have multiple quarters harboring 1 or 2 *S. haemolyticus* RAPD types, while cow C, J, and K had multiple teat apices positive for one or two RAPD types (Figure 5). In herd 5, *S. haemolyticus* RAPD type c (*n* = 12) was found in quarter milk both with low SCC or clinical signs, teat apices, and rectal feces (Figure 6). Cow C and E had more than one teat apex positive for RAPD type c. Cow D has 2 teat apices positive for either RAPD type a or c.

**Figure 7:**
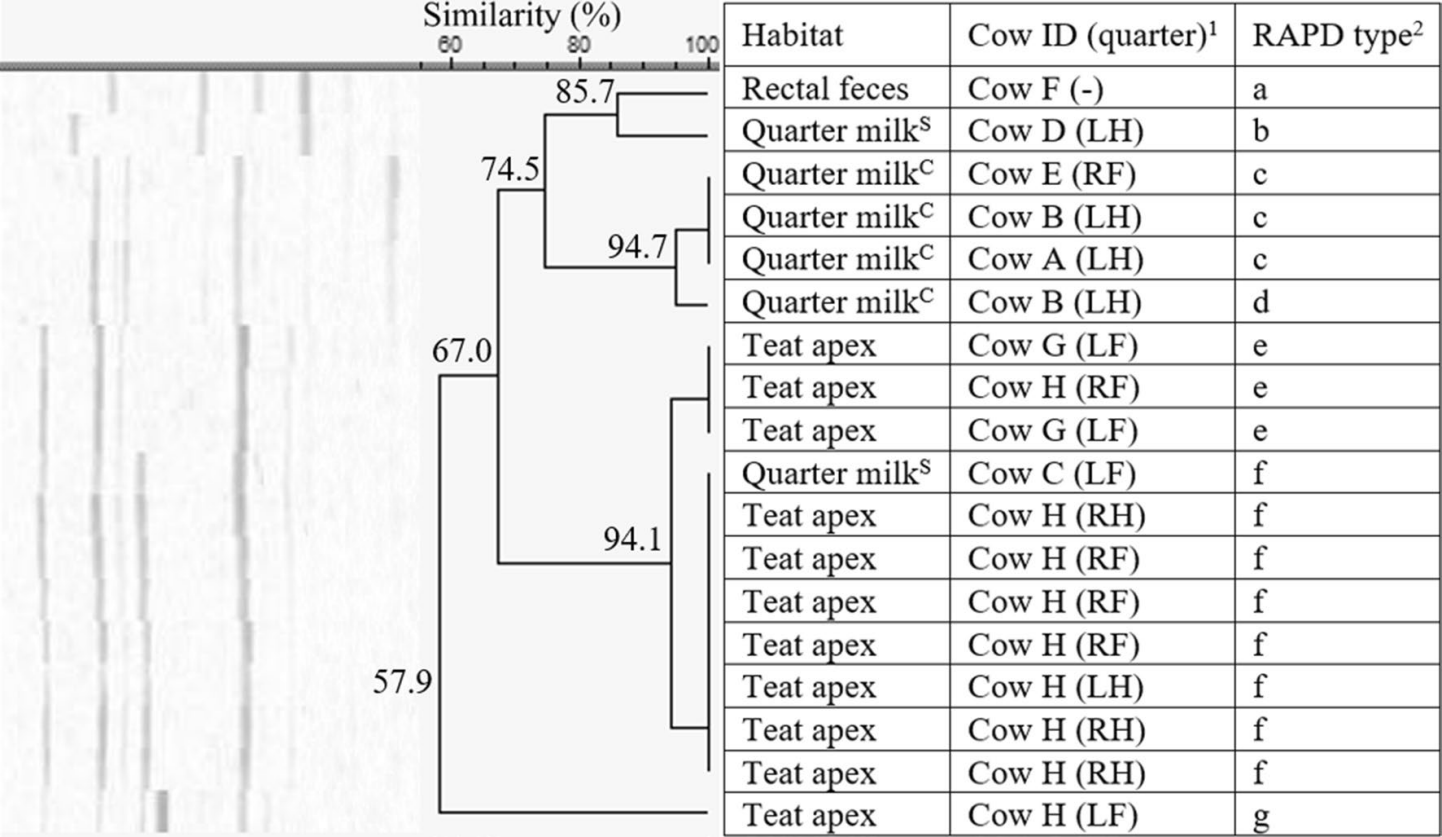
**Dendrogram of the RAPD fingerprints of 18*Staphylococcus hominis*isolates, originating from herd 1.**^1^Cows were assigned an arbitrary upper case letter, independently of the other dendrograms. ^2^RAPD types were assigned an arbitrary lower case letter based on the clustering. ^H^Quarter milk samples with a SCC ≤ 50 000 cells/mL milk (healthy). ^S^Quarter milk samples with a SCC > 50 000 cells/mL milk (subclinical mastitis). ^C^Quarter milk samples from a quarter with clinical signs of mastitis (clinical mastitis).

**Figure 8:**
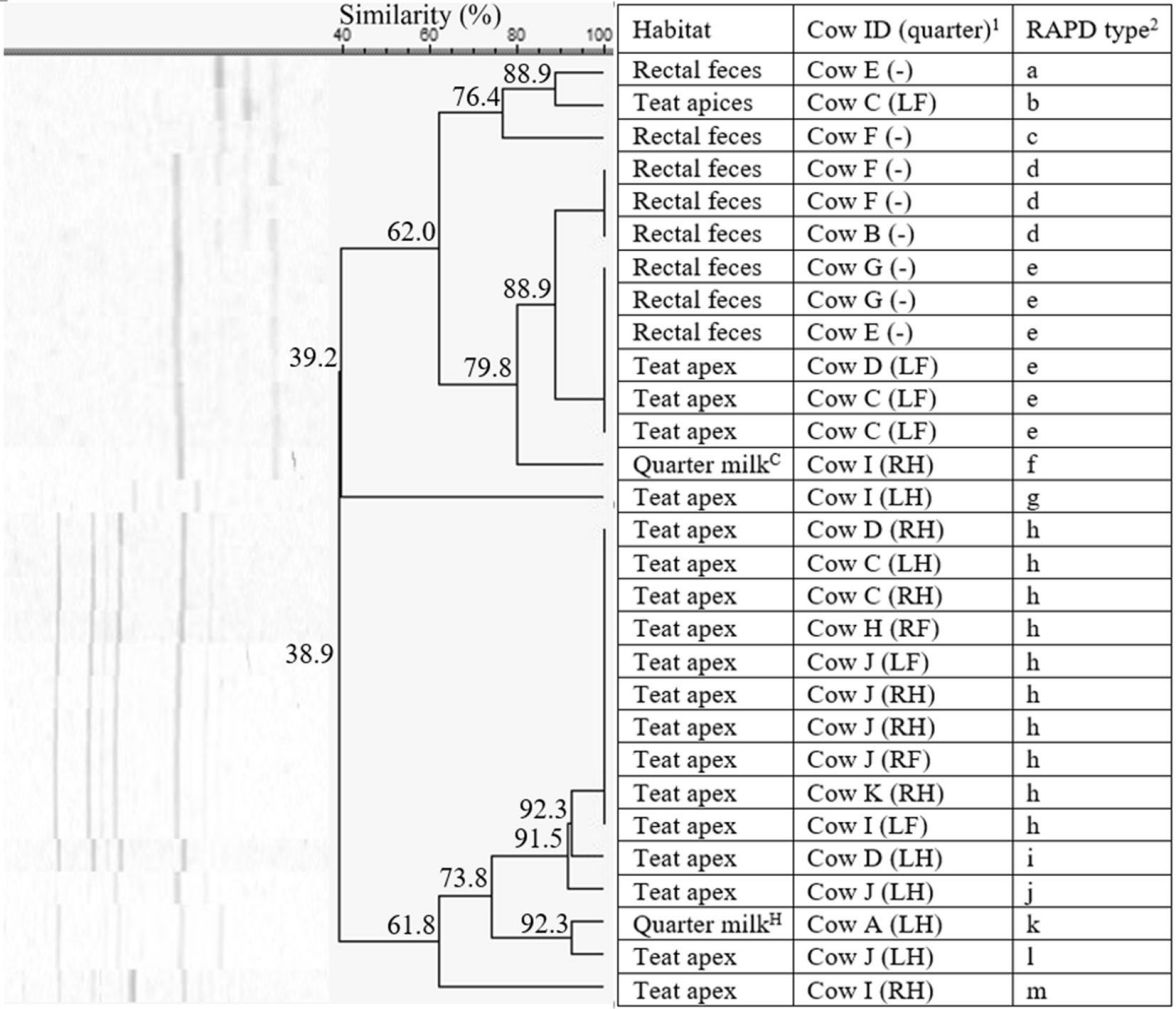
**Dendrogram of the RAPD fingerprints of 29*Staphylococcus hominis*isolates, originating from herd 2.**^1^Cows were assigned an arbitrary upper case letter, independently of the other dendrograms. ^2^RAPD types were assigned an arbitrary lower case letter based on the clustering. ^H^Quarter milk samples with a SCC ≤ 50 000 cells/mL milk (healthy). ^S^Quarter milk samples with a SCC > 50 000 cells/mL milk (subclinical mastitis).

**Figure 9:**
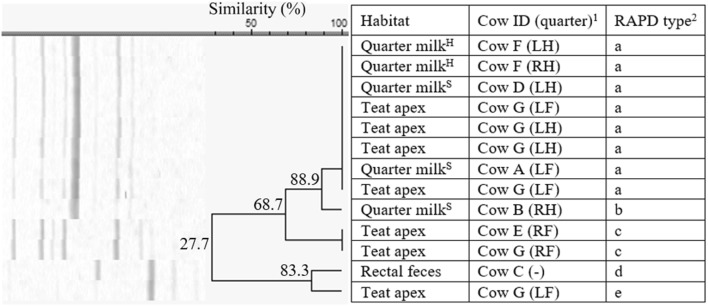
**Dendrogram of the RAPD fingerprints of 13*Staphylococcus hominis*isolates, originating from herd 3.**^1^Cows were assigned an arbitrary upper case letter, independently of the other dendrograms. ^2^RAPD types were assigned an arbitrary lower case letter based on the clustering. ^H^Quarter milk samples with a SCC ≤ 50 000 cells/mL milk (healthy). ^S^Quarter milk samples with a SCC > 50 000 cells/mL milk (subclinical mastitis). ^C^Quarter milk samples from a quarter with clinical signs of mastitis (clinical mastitis).

**Figure 10:**
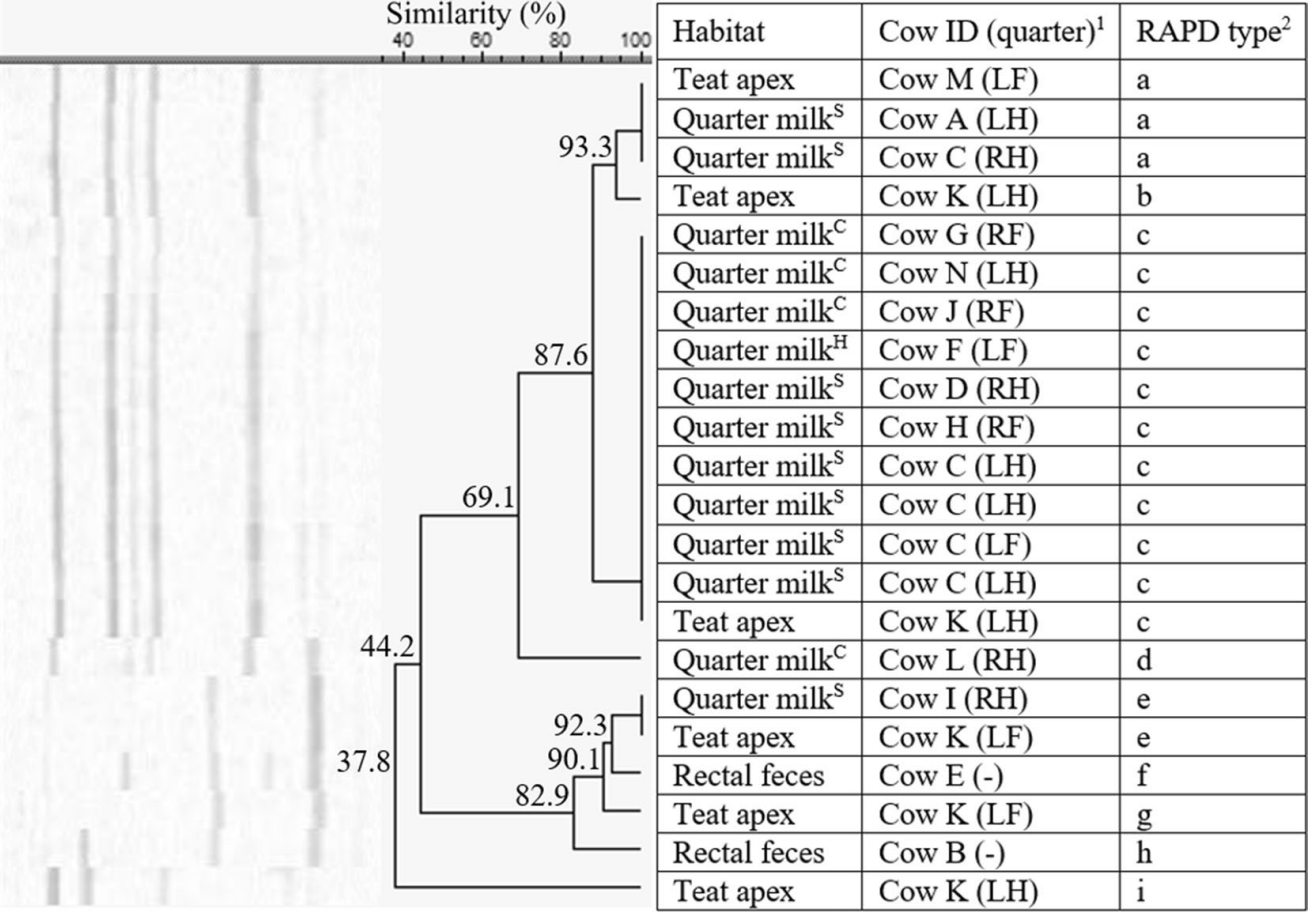
**Dendrogram of the RAPD fingerprints of 22*Staphylococcus hominis*isolates, originating from herd 4.**^1^Cows were assigned an arbitrary upper case letter, independently of the other dendrograms. ^2^RAPD types were assigned an arbitrary lower case letter based on the clustering. ^H^Quarter milk samples with a SCC ≤ 50 000 cells/mL milk (healthy). ^S^Quarter milk samples with a SCC > 50 000 cells/mL milk (subclinical mastitis). ^C^Quarter milk samples from a quarter with clinical signs of mastitis (clinical mastitis).

The 82 *S. hominis* isolates were found in quarter milk, teat apices, and rectal feces on all but herd 5 (Figures 7, 8, 9, 10). In herd 1, type c, e, and f were identified more than once, whereas this were type d, e, and h, type a and c, and type a, c, and e in herd 2, herd 3, and herd 4, respectively. The isolates were divided in 5 to 13 different RAPD types in the 4 herds with a similarity of 57.9%, 38.9%, 27.7%, and 37.8% in herd 1, 2, 3, and 4, respectively. The *S. hominis* isolates originating from rectal feces belonged to 1, 4, 1, and 2 RAPD types, on herd 1, 2, 3, and 4, respectively. Seven of those 8 types were habitat-specific, only RAPD type e from herd 2 consisted of isolates from both teat apices and rectal feces. In herd 1, RAPD type c and e were found in either quarter milk from clinical mastitis or teat apex samples only. The left hind quarter milk sample of cow B was positive for both RAPD type c and d. Cow H had one or two *S. hominis* types on all 4 teat apices. In herd 2, RAPD type h was recovered from teat apices only. Cow B, E, F, and G, had one or 2 RAPD types in the rectal fecal samples. Cow C, D, I, and J had 3 or 4 teat apices either positive for one or 2 RAPD types. Type a from herd 3 and type c from herd 4 were was recovered both from quarter milk and teat apices. As tabulated in Table 2, Cow G (herd 3) and cow K (herd 4) had one to 3 *S. hominis* RAPD types on multiple teat apices.

## Discussion

This is the first study investigating whether fecal NAS are potential causes of bovine IMI, using RAPD-PCR. For *S. chromogenes*, *S. devriesei*, and *S. haemolyticus* (herd 5), the same RAPD fingerprints were found in rectal feces, teat apices, and quarter milk suggesting that NAS originating from rectal feces can cause IMI. Although the teat apex and teat canal form the main entryway to the udder for microorganisms [19], some authors suggest the existence of a so-called entero-mammary pathway [20]. Transfer of bacteria from the gut via intracellular transport to the mammary gland has been proven for mice and suggested for humans [21–23] yet the immune system of the mammary gland and gut is not as closely linked in ruminants as in monogastric species [24] making the hypothesis that NAS (or other bacteria) can reach the mammary gland without entering through the teat canal less likely [25]. A study specifically designed to test this hypothesis, would require the confirmation of the transfer of viable bacteria from the gut to peripheral lymph nodes and mammary gland via (migrating leukocytes in) the lymphatic and/or blood circulation. For *S. cohnii*, the same strains present in rectal feces and on teat apices were only found in milk samples from healthy quarters in the absence of inflammation. Either these strains should be seen as commensals, or the milk samples were contaminated when samples were taken [26].

For *S. hominis*, *S. equorum*, and *S. haemolyticus* (herd 1), we were unable to confirm the presence of the same strains in the three different habitats. This could reflect species differences or be due to the selection criteria and limited number of RAPD typed isolates per NAS species. Per herd only a random selection of lactating cows was sampled and each cow was sampled once for either quarter milk, teat apices, or rectal feces. Also, not all isolates originating from the teat apices were included in the RAPD analysis which could have influenced our results as well.

The diversity degree for *S. chromogenes* in this study is comparable to the genetic diversity reported by others [27–29], although a lower degree of diversity was presented by Shimizu et al. [30] and Piessens et al. [31]. For *S. cohnii,* high similarity scores were found indicating a low genetic diversity unlike prior research [29]. The diversity for *S. devriesei* is comparable to the findings of Supré et al. [32]. For *S. equorum,* a wide genetic diversity has been previously described for isolates originating from sausages and their environment [33]. Our findings corroborate with earlier studies that reported *S. haemolyticus* and *S. hominis* are highly diverse species and adaptable to various conditions [12, 29, 31]. The differences in reported diversity between studies can also be explained by the limited number and origin of isolates and the used techniques.

For *S. haemolyticus* (herd 1) and *S. hominis,* fecal isolates might be more adapted to survival in rectal feces than in quarter milk or on teat apices as only 1 out of 8 RAPD types divided over 4 herds was retrieved from both teat apices and rectal feces. For *S. chromogenes*, *S. devriesei*, *S. equorum*, *S. haemolyticus* (herd 5), and *S. hominis,* one or more strains were solely found in either quarter milk or on teat apices suggesting some strains within species could be more udder-adapted while others have no or a lower ability to colonize the mammary gland. Strain differences within NAS species have actually been confirmed before. E.g. we reported that *S. chromogenes* originating from a persistent IMI displays a better in vitro bacterial growth in conditions mimicking the mammary gland and in vivo bacterial growth in the mammary glands of mice and dairy cows, compared to *S. chromogenes* isolated from the teat apex [9, 34, 35]. Differences in inflammatory response in both mice and dairy cows between strains of the same species and growth inhibition of other major mastitis pathogens have been demonstrated as well [35, 36], indicating it is relevant to subtype NAS in future research efforts.

Some NAS species tend to be more relevant for the udder health, e.g. *S. chromogenes* [8, 37, 38]. This was substantiated in the current study as in herd 1, only milk samples from quarters with a SCC > 50 000 cells/mL milk and clinical signs harbored *S. chromogenes*. Also, the same strain was isolated from a quarter with subclinical mastitis and a quarter with clinical signs, substantiating that not only the virulence potential of the pathogen but also host factors might influence the severity of mastitis. *Staphylococcus hominis* was reguraly isolated from quarters with subclinical and clinical mastitis in herd 1 and 4. These results deviate from those reported by Jenkins et al. [29] and Condas et al. [39]. While *S. cohnii* was found more often in healthy quarters (SCC ≤ 50 000 cells/mL milk) which is in agreement with Supré et al. [37], suggesting a potential commensal nature of this species or sample contamination as *S. cohnii* was also isolated from one clinical mastitis case.

Random amplification of polymorphic DNA is a rapid and easy to perform technique that is commonly used for comparing bacterial communities with consistent results compared to other techniques such as restriction fragment length polymorphism, amplified fragment typing methods, and pulsed-field gel electrophoresis (PFGE) [40–43]. Still, it is known that RAPD can lack some discriminatory power for some species [31]. For specific species, e.g. *S. haemolyticus* and *S. simulans*, the combination with other techniques such as PFGE and amplified fragment typing methods could improve the detection of variation between isolates [31, 44]. Still, it is hard to compare fingerprint-based methods between laboratories [45]. The low mutation rate of housekeeping genes makes multilocus sequence typing more suitable, but it is currently only available for *S. epidermidis*, *S. haemolyticus*, *S. hominis*, *S. lugdunensis*, and *S. pseudintermedius* [46–50]. Whole genome sequencing provides a theoretically optimal resolution, but is, momentarily, less suitable for larger collections of isolates and routine analyses [51].

For *S. chromogenes*, *S. cohnii*, *S. devriesei*, and *S. haemolyticus*, the same RAPD type was found in rectal feces, teat apices, and quarter milk originating from the same herd, suggesting fecal NAS can infect the bovine mammary gland, at least for some species. For *S. hominis* and *S. equorum*, we were unable to confirm the presence of the same RAPD strain in the three habitats.

## Data Availability

The data on which the conclusions of the manuscript rely are presented in the main paper.

## Abbreviations

DNA: deoxyribonucleic acid
IMI: intramammary infection
MALDI-Tof: matrix-assisted laser desorption/ionization time-of-flight mass spectrometry
MSA: mannitol salt agar
*n*: number
NAS: non-aureus staphylococci
PFGE: pulsed-field gel electrophoresis
RAPD-PCR: random amplification of polymorphic deoxyribonucleic acid polymerase chain reaction
*S.*: *Staphylococcus*
SCC: quarter somatic cell count
